# Linking Myometrial Physiology to Intrauterine Pressure; How Tissue-Level Contractions Create Uterine Contractions of Labor

**DOI:** 10.1371/journal.pcbi.1003850

**Published:** 2014-10-16

**Authors:** Roger C. Young, Peter Barendse

**Affiliations:** 1 Department of Obstetrics and Gynecology, University of Tennessee Health Science Center, Memphis, Tennessee, United States of America; 2 Wolfram Research, Somerville, Massachusetts, United States of America; Washington University in St. Louis, United States of America

## Abstract

The mechanisms used to coordinate uterine contractions are not known. We develop a new model based on the proposal that there is a maximum distance to which action potentials can propagate in the uterine wall. This establishes “regions”, where one action potential burst can rapidly recruit all the tissue. Regions are recruited into an organ-level contraction via a stretch-initiated contraction mechanism (myometrial myogenic response). Each uterine contraction begins with a regional contraction, which slightly increases intrauterine pressure. Higher pressure raises tension throughout the uterine wall, which initiates contractions of more regions and further increases pressure. The positive feedback synchronizes regional contractions into an organ-level contraction. Cellular automaton (CA) simulations are performed with Mathematica. Each “cell” is a region that is assigned an action potential threshold. An anatomy sensitivity factor converts intrauterine pressure to regional tension through the Law of Laplace. A regional contraction occurs when regional tension exceeds regional threshold. Other input variables are: starting and minimum pressure, burst and refractory period durations, enhanced contractile activity during an electrical burst, and reduced activity during the refractory period. Complex patterns of pressure development are seen that mimic the contraction patterns observed in laboring women. Emergent behavior is observed, including global synchronization, multiple pace making regions, and system memory of prior conditions. The complex effects of nifedipine and oxytocin exposure are simulated. The force produced can vary as a nonlinear function of the number of regions. The simulation directly links tissue-level physiology to human labor. The concept of a uterine pacemaker is re-evaluated because pace making activity may occur well before expression of a contraction. We propose a new classification system for biological CAs that parallels the 4-class system of Wolfram. However, instead of classifying the rules, biological CAs should classify the set of input values for the rules that describe the relevant biology.

## Author Summary

How does the pregnant uterus coordinate a kilogram of smooth muscle tissue into repetitive, synchronous, organ-level contractions of human labor? Action potential propagation recruits tissue for contraction over short distances, but a single action potential sweeping through the uterus cannot explain organ-level function. Multiple action potentials seem to arise simultaneously, and apparently spontaneously, in different “regions” of the uterine wall. We interpret the existence of regions as a consequence of there being a maximum distance a single action potential can travel. To explain the synchronization of regional contractions, we use a second mechanism: initiation of contractions by stretch. Because the uterus is pressurized, contraction of the first region raises the intrauterine pressure slightly, which stretches the entire uterine wall. The stretch recruits another regional contraction, which generates more pressure. This positive feed-back recruits most regions into simultaneous activity. With this mechanism we simulate the contraction patterns of human labor, and show how contractions emerge from complex interactions. We explain why the decades-long search for the uterine pacemaker has failed, and why drugs that stimulate or inhibit tissue contractions have enigmatic effects at the organ-level. This simulation, for the first time, successfully links tissue experiments to clinical obstetrics.

## Introduction

Over the past several decades, a large body of literature has addressed intracellular [1] and intercellular [2] signaling in pregnant myometrium, but comparatively little effort has been spent seeking to describe the mechanisms that coordinate contractions at the level of the whole uterus. It is clear that at the cellular level, electrical excitability properties control both the signal to contract and the mechanism to directly raise intracellular calcium. Tissue recruitment by action potential propagation is dominant at the tissue-level [2], [3] (in the order of centimeters), and it is generally believed to play a key role at the organ-level in human labor [4], [5] (in the order of several 10 s of centimeters).

However, it remains unclear how the pregnant uterus coordinates a kilogram of myometrium into repetitive, synchronous contractions of normal human labor. Several mathematical simulations of organ-level functioning have been published, including action potential propagation mechanisms alone [6], [7], calcium waves [8], and a previous cellular automaton approach [9]. Each has significant limitations, including failure to predict uterine behavior across the spectrum of clinical function, or direct disagreement with subsequently published data [10]. These limitations seem to reveal a poor understanding of how the uterus communicates at the organ-level.

In the canonical action potential propagation model, an action potential originates at a pacemaker site, travels through the wall of the entire uterus, exciting and recruiting tissue as it propagates [4], [5]. Despite being widely accepted, there is no direct evidence to suggest that organ-level recruitment utilizes a stable pacemaker site or a single propagating action potential. Indeed, there is good evidence to the contrary. The highest resolution electrical mapping of the pregnant uterus was reported by Lammers et al. in the guinea pig [11]. They found that the first spike of the action potential rarely activated the entire field. A given action potential burst seemed limited to about 10 cm^2^, and burst propagation speeds were much slower than action potential propagation speeds. Additionally, a stable pacemaker was not identified, and the pathways of propagation were described as “tortuous”.

The only high resolution mapping of the entire front of the human uterus are by Ramon [12] and Eswaran [13], using an array of superconducting quantum interference devices (SQUID). Synchronization analysis suggested that the human uterus is similar to the guinea pig uterus, as there appears to be a maximum distance of propagation for an action potential. Specifically, wave fronts of electrical activity that propagated long distances were not observed. Although recurrent electrical activities tended to occur in the same physical locations, they also failed to observe a stable pacemaker. These observations raise the possibility that despite its irrefutable importance at the tissue-level, a mechanism using only action potential propagation for tissue recruitment is insufficient, and a second mechanism may be functioning at the organ-level.

In 1970 the action potential propagation model was the leading candidate for organ-level signaling, but Csapo [14] proposed an alternate mechanism - mechanotransduction by pressure-tension sensing. In brief, he proposed that increases of intrauterine pressure cause increases of wall tension (per the Law of Laplace; T = P*r/w), which then initiate contractions throughout the uterus. Unfortunately, the way mechanotransduction was initially presented suggested that contractions were controlled by stretch, and that electrical activity, if present at all, played a minimal role in initiating contractions or recruiting tissue. This seemed to present a choice between two mutually exclusive mechanisms. Over the next several years it was established without question that electrical activity causes contractions [3], and expression of an action potential is necessary and sufficient for tissue-level contractions [15]. Thus, the action potential propagation hypothesis was viewed as the winner, and over time, mechanotransduction was largely forgotten.

However, given the difficulty action potential propagation alone has explaining organ-level function, we revisited Csapo's mechanotransduction mechanism. It is has long been known that quickly stretching smooth muscle can initiate a contraction, and myometrium is no exception. In the 1970s, however, Csapo was perhaps unaware that acutely stretching, or increasing tension on myometrium, initiates an action potential burst which contributes to at least part of the underlying mechanism of stretch-initiated contractions [16]. Hence, mechanotransduction and action potential propagation are complementary and not mutually exclusive.

In this work we will use the term “mechanotransduction” primarily to refer to the mechanism, and “stretch-initiated” to refer to the phenomenon. In the discussion we will explain what we mean by the phrase “myometrial myogenic response”, which, we suggest, emphasizes the importance of stretch-initiated contractions to normal function. This term is also intended to highlight the similarity of myometrial and vascular smooth muscle functioning, and imply that the cellular mechanisms may overlap. The arterial myogenic response is understood in fair detail [17], but the physiological processes following acute stretch of myometrium are only superficially understood.

To build the model that incorporates both action potential propagation and mechanotransduction, we make one key assumption. That is, there is an upper limit on the distance each tissue-level action potential can travel. If true, this limitation defines functional regions of the uterine wall. The SQUID array synchronization analysis provides some evidence for the existence of these regions, and an approximate size of 8 cm×8 cm (reference 12, figure 8). Within regions, we assume, tissue recruitment is entirely by action potential propagation.

To coordinate regional contractions into organ-level contractions, we use mechanotransduction as follows: If one region contracts, the intrauterine pressure rises slightly. This pressure rise would increase tension on the other areas of the uterine wall according to the Law of Laplace (T = P*r/w). Increasing tension could trigger a stretch-initiated contraction of another region. With two regions contracting, pressure would increase further, and the cycle would repeat until all regions capable of expressing a stretch-initiated contraction were recruited.

A key feature of this mechanism is that it is a pressurized hydraulic system, similar to what is used for automobile braking. Hydraulic systems rapidly transmit signals over long distances, and are not limited by physical proximity to the event that initiates the signal. Therefore, because pressure is the signal used to recruit regions, coordinating a uterine contraction at the organ-level is not constrained by the speed of action potential propagation, even though tissue within regions may be wholly recruited by action potentials.

Here we report a mathematical simulation of our model for creating uterine contractions of human labor. We use a variation of the cellular automaton (CA) technique. A CA is a system of “cells”, each of which has a state that is defined by the state of the other cells through one or more “rules”. As detailed above, our model divides the uterus into regions, where a cell represents each region (e.g. “cell” does *not* refer to an individual myocyte). With each iteration, or time step, the status of all the cells are simultaneously updated. Classical, or elemental CAs apply one rule and consider only the state of each cell's near neighbors. Complex biological CAs use multiple rules to describe complex physiology, and the status of a cell may be influenced by cells that extend beyond the neighbors.

## Methods

### The physiological basis of the rules

**Figure 1:**
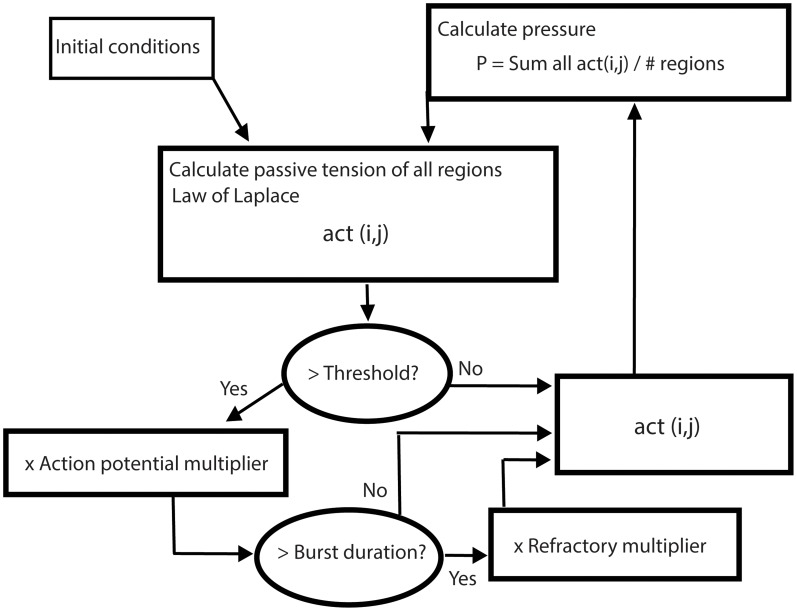
Flowchart of cellular automaton simulation.

For human labor, the contractile state of the uterus and the intrauterine pressure are of primary concern. Since the intrauterine pressure is shared by all regions of the uterine wall regardless of relative location, we weight the nearest neighbors no more or less than the other regions. The CA rules approach to simulation is computationally efficient but, more importantly, emphasizes the physiological properties of the tissues that make up the organ. With this in mind, we define three specific rules (see flowchart figure 1): 1) Intrauterine pressure at each time step is calculated as a function of the regional contractile activities. 2) At the next time step, the intrauterine pressure sets the passive tension on each region according to the Law of Laplace. 3) Within regions, electrical activity creates contractile activity (defined as the tension of a contraction). The tension on each region will initiate and maintain an action potential burst if it exceeds a defined threshold (action potential threshold). By definition, if any part of a region experiences an action potential, the action potential travels throughout the entire region, but no farther, and the region contracts as a unit. When a region is experiencing an action potential burst, the contractile activity is calculated by multiplying the passive tension by the action potential multiplier (a factor >1). Each region can remain electrically active no more than a defined number of time steps (burst duration). If a region has been electrically active for the maximum allowable number of time steps, it enters a refractory period. When a region is in the refractory period, the tension is decreased by multiplying the passive tension by a factor <1 (refractory multiplier). The refractory period lasts a defined number of time steps (refractory duration – not shown in figure 1), then the region reverts back to expressing the passive tension.

### Rule 1. Calculating intrauterine pressure from regional contractile activities

**Figure 2:**
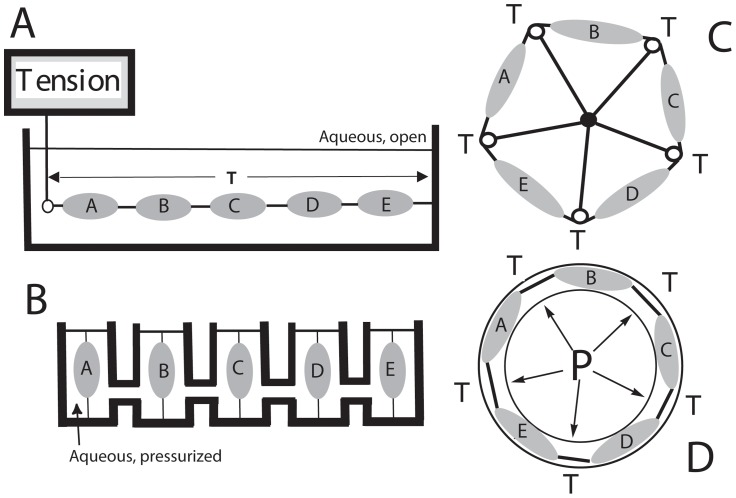
Multi-tissue, isometric contractility thought experiments. A) Isometric arrangement with tissues A–E in series. B) The hydrodynamic pressure chamber equivalent of A, where all chambers communicate through fluid filled channels. Tissues are rigidly attached below, but attached to a moveable piston at the top. C) The tissues are isometrically arranged in a circle, in analogy with A. Pulleys are between tissues, and the pulleys are supported by rigid bars. D) A schematic of the human uterus in cross section. Intrauterine pressure replaces the pulleys and rigid bars of C, and the hydrodynamics correspond with B. In both B and D, each tissue senses the same pressure.

In figure 2A we represent a hypothetical isometric contractility experiment, except that instead of a single tissue strip, tissues A through E are mechanically linked end-to-end. The total tension on the system is T, which is the same for each tissue. If any tissue contracts, T will increase and the new T will be expressed equally across each tissue. In figure 2B we present the corresponding hydrodynamic experiment. Here there are 5 hydraulically connected identical chambers, each containing one tissue strip that is fixed at the bottom and attached to a moveable piston at the top. The entire volume is filled with an incompressible medium, because under these conditions, the volume of the system is constant. For the human uterus, this is an excellent assumption since air is never seen within the uterine cavity, even after rupture of membranes. In this hypothetical apparatus, contraction and shortening of one tissue would pull the piston downward and increase pressure throughout the system. In the chambers with the 4 non-contracting tissues, the pistons would move outward, and the stretching would increase tension on each tissue. While somewhat counterintuitive, the two arrangements in figure 2A and B are mechanically identical. Translating these models to the physical structure of the gravid uterus, figure 2C represents the “tension” descriptor that is a wrap-around of figure 2A, and figure 2D represents a Law of Laplace interpretation that corresponds to both figures 2B and C.

Consider the hydraulic system in figure 2B in detail. Because of the viscoelastic properties of myometrium, the system is in hydraulic equilibrium at only two times – when no contractions are occurring, and when all tissues are maximally contracting simultaneously. At these times Pascal's Law applies, and P = Force/area in all chambers. This equation does not apply during the onset and offset of the contraction because of the viscoelastic properties of the tissues undergoing passive stretch.

Assuming nearly identical tissues and identical piston areas, the peak pressure throughout the system when all the tissues are contracting simultaneously is equal to the peak pressure (P_max_) generated by one tissue in an isolated chamber. When no tissues are contracting, the minimum pressure equals the baseline tension, which is equally expressed on each tissue. If one tissue contracts, the pressure begins to rise in all the chambers. The elasticity of the four tissues that are not contracting slows the rate of pressure development. If another tissue contracts, the elasticity of that tissue is lost, and pressure rises faster. Therefore, we will approximate the system pressure at any time as linearly proportional to the average contractile activity of the regions.

Applying this reasoning to the five chamber experiment in figure 2B, if the first tissue contracts maximally, the system pressure will rise to approximately 20% P_max_. The pistons in the other chambers move outward in response to the rising pressure, and the non-contracting tissues are stretched. A stretch-initiated contraction occurs if one of the four remaining tissues is susceptible. Because of hydraulic signaling, *the susceptible tissue that contracts next does not need to be physically located adjacent to the tissue that contracted first*. With two tissues contracting maximally, the pressure increases to ∼40% P_max_, which further pushes the pistons of the remaining three tissues. With the additional stretch, the next most susceptible tissue may then contract, and so on, until all tissues contract simultaneously and the system pressure equals P_max_.

Because tissue contractions are caused by electrical activity, in the simulation we will use the term “activity” to mean both electrical activity and contractile activity of a region. In addition, viscoelastic tissue creates tension passively when stretched, and we will use the term “passive activity” or passive tension specifically as the force generated by a tissue that is not caused by contractile activity.

Using this nomenclature:

Pressure(t) = ∑activity of all regions(t)/#regions

### Rule 2. The Law of Laplace and the importance of local anatomic variations

In a closed container containing an incompressible fluid, pressure (P) and wall tension (T) are related by the Law of Laplace:

T = P*r/w

The r/w factor accounts for the physical shape of the container, or in this case, the anatomy of the uterus. w is the thickness of the wall and r is the local radius of curvature. r can be measured in perpendicular x,y axes, where 1/r = 1/r_x_+1/r_y_. If r_x_ = r_y_, a factor of 2 is introduced in the denominator, making the Law of Laplace for a sphere. If r_x_ is infinity, r = r_y_, and it becomes the Law of Laplace for a cylinder.

Differences of local r and w mean that there are regional variations due to local uterine anatomy that must be accounted for when calculating either T from P, or P from T. We introduce the “anatomy sensitivity factor” to account for these variations. Each region has its own multiplicative factor corresponding to r/w, and we will assume that it is the same for the entire region, and that it does not significantly change for the duration of the run.

For the gravid uterus (an oblate spheroid), the largest r_x_ will be close to the “sphere radius” of the uterus, or ∼10 cm, but as short as a few cm at regions of high curvature. Thus, r_x_ conservatively ranges over about a factor of 3. w varies by approximately a factor of 2 [18], and the sphere-cylinder difference (r_x_ may be very large for an oblate spheroid) brings in another factor of 2 that associates with w. Using these approximations, the anatomy sensitivity multiplier will vary from 1/4 to 3/1 (range of the numerator/range of the denominator). This results in a skewed distribution of this factor between.25 and 3, with most values near 1. To simulate this, we define the anatomysens(i,j) matrix. Each (i,j) value of the matrix is the anatomy sensitivity multiplier of the (i,j) region, and it is held constant through each run of the simulation. To convert from pressure to the passive activity of the (i,j) region, we multiply by anatomysens(i,j). When pressure is calculated from the regional activities, we will divide by the same factor. To set specific values between ∼.25 and 3 centered around 1, we select pseudorandom numbers from a Weibull distribution. This distribution is shaped by three parameters, the first sets the shape, the second the scale, and the third establishes the starting location.

Hence,

act(i,j) = pressure * anatomysens(i,j)

and

pressure = ∑(act(i,j)/(#regions * anatomysens(i,j))

where act(i,j) is the contractile activity of the i,j region at a specific time step. Since act(i,j) varies over time, the time series is stored in the activity tensor, activity(i,j,t).

### Rule 3. Accounting for electrical activity within and among regions

SQUID data suggest that repetitively active regions maintain the same location, at least on the time scale of the reported experiments (several contractions). Hence, we will also assume the regions are physically stable over the duration of each run of the simulation.

When stretched, each region will either increase tension passively, or it may contract if a propagating action potential is initiated. To simulate a stretch-initiated contraction, we assign each region a tension threshold value. An active contraction occurs if the wall tension of a region exceeds its threshold. To emphasize a key element of our model - that propagating action potentials recruit tissue within regions - we will use the more specific term “action potential threshold” to refer to the value of the tension that initiates a contraction.

It is unlikely that the action potential threshold is the same for all the regions. Since this is possibly a key factor in the onset of labor, we will again allow flexibility in the simulation by pseudorandomly selecting action potential threshold values from a Weibull distribution. When a regional tension exceeds that region's action potential threshold, we simulate the contraction by multiplying the passive tension by the action potential multiplier (an input variable >1).

From isometric muscle bath experiments [19], it is well-understood that tissue relaxation begins when the burst stops, and there appears to be an upper limit to the duration of a burst. In our simulation, once a regional burst is initiated, it will remain on until either the activity falls below threshold, or the tissue reaches its maximum burst duration (an input variable). We therefore calculate the (i,j,t) burst tensor such that each region experiences independent bursting behavior as follows:

If act(i,j) is below threshold, then burst(i,j) = 1.

If act(i,j) is above threshold, then bursting occurs and burst(i,j) = action potential multiplier.

However, if the Maximum burst duration is exceeded, then burst(i,j) = 1

Immediately following a burst, the tissue relaxes and it is relatively unresponsive to initiation of another contraction. To simulate this refractory period, we apply the refractory multiplier (input variable, value <1). The refractory period has a defined refractory duration (input variable).

If the Maximum burst duration is not exceeded, then refractory(i,j) = 1.

If the Maximum burst duration is exceeded, then refractory(i,j) = refractory multiplier.

It the Refractory duration is exceeded, then refractory(i,j) = 1.

Thus, rule 3 corrects each region's contractile activity by the burst and refractory matrices during each time step of the simulation.

act(i,j) = act(i,j)×burst(i,j)×refractory(i,j)

### Additional details of the calculations

1. To visualize this simulation, we imagined the uterine wall as being composed of similarly sized hexagons, like a soccer ball. The orientation of the display of the regions is arbitrary, although conversationally we visualize opening the uterus from a point in the center of the back and orienting the cervix downward. Because we are omitting near neighbor effects, we can envision all connections between the tiles opened and the tiles laid flat. For display, spaces between the tiles are removed, and the hexagons are converted to squares. A term gravid uterus contains 4.5 kg of fetus, placenta, and amniotic fluid. The radius of a sphere containing 4.5 L is 10.2 cm and has a surface area in the order of 1300 cm^2^. The SQUID synchronization data of Ramon [12]) suggest regions are ∼8 cm×8 cm, or 64 cm^2^. Thus, if the uterus were a sphere there will be 20 to 21 regions. For an oblate spheroid of the same volume, there will be approximately 25–30 regions.
2. Simulations were programed using Mathematica (version 9). See Supporting Information S1 for the Mathematica program file and Supporting Information S2 for a PDF of the program. The regions of the uterus are represented as an array of i rows and j columns. Thus, a 5×5 array would represent 25 functional regions. This simulation accepts from 1 to 64 regions. We assume a constant volume and a uterine wall surface area that changes only slightly in response to pressure increases. Therefore, increasing the number of regions reduces the size of the regions. Since the distance an action potential can propagate sets the region size, increasing the number of regions simulates reducing the propagation distance of the tissue-level action potential. For 64 regions, and an oblate spheroid (area ∼1900 cm^2^) the size of each region is approximately 5.5 cm×5.5 cm. Using 9 regions, the size of each region is approximately 14.5 cm×14.5 cm, and the physical arrangement is roughly equivalent to assuming the front half of the uterus is divided into quadrants,
3. Within regions, tissue is recruited via electrical activity. Since action potentials have been shown to propagate at speeds in the order of 3 cm/sec, the time required to recruit all the tissue within each region is <5 seconds for 9 regions, and ∼2–3 seconds for 16 or more. Since uterine contractions typically last 60 seconds or more, we assume that tissue recruitment within each region occurs much more rapidly than organ-level recruitment of regions. Additionally, we will use simulation step times that correspond to ∼5 seconds (see #6 below). In this manner we can assume that the regions expresses uniform activity.
4. For purposes of colorization and visual display of simulation output, act(i,j) was allowed to range between 0 and 10, with 0 being fully relaxed and 10 fully contracted. Because of variations of the anatomy sensitivity matrix for a specific run, the maximum pressure generated by an organ-level contraction varied between approximately 8 and 12.
5. Because we do not know the true distribution for either the anatomic sensitivities or the action potential thresholds, we pseudorandomly select numbers from a statistical distribution. Once established, the values are not changed during a run. In Mathematica, the specific values selected from the distributions can be changed by selecting a different seed. The advantage of this method is that by using the same seed, simulations can be compared without changing the distributions. We use the Weibull distribution rather than Gaussian because of ease of adjusting the skewedness and the lower limit of the curve.
6. Experimental data show that electrical bursts last up to 50–60 seconds in human myometrial strips. By selecting the maximum burst duration to be 10–12 iterations, each step then represents in the order of 5 seconds.
7. Intrauterine pressure catheter measurements demonstrate that the human uterus in labor maintains a pressure between contractions of approximately 15 torr, and the highest pressure that can be generated by the term gravid uterus is approximately 300 torr. Therefore, the pressure between contractions (which we call the minimum pressure) is ∼1/20 maximal pressures. In the simulation we allow the minimum pressure to be changed, but with a value of ∼10 for the maximum pressure, minimum pressure should remain near 0.5 to be physiologically reasonable.
8. Our hydrodynamic model is based on Pascal's principle, which states that pressure equilibrates very rapidly within a pressurized system (essentially at the speed of sound). Using this principle, regions can signal to each other instantaneously over very long distances. The most novel element of the model - that physical proximity does not influence recruitment of regions - is a direct result of Pascal's principle.

### Formal presentation of the rules of the simulation

pressure(t) = ∑act(i,j)/(# regions * anatomysens(i,j))

at the next time step:

act(i,j,t+1) = pressure(t) * anatomysens(i,j) * burst(i,j) * refractoryfactor(i,j)

 where

burst(i,j,t+1) = 1, if act(i,j,t+1)<threshold(i,j).

burst(i,j,t+1) = action potential multiplier, if act(i,j,t+1)>threshold(i,j).

burst(i,j,t+1) = 1, if duration of the burst>max burst duration.

refractoryfactor(i,j,t+1) = 1, if duration of the burst< = max burst duration.

refractoryfactor(i,j,t+1) = refractory multiplier, if duration of the burst>max burst duration.

refractoryfactor(i,j,t+1) = 1, if refractory period duration>max refractory duration.

The names and descriptions of the input, calculation, and output variables are in Table 1. Because there are a number of input variables, we have selected the following short-cut descriptors to allow changing conditions to be easily compared:

**Table 1:** Summary of variables.

Input variables	Function (variable name used in program)
rows	# rows (rows)
columns	# columns (columns)
time steps	# time iterations used for the simulation (timesteps)
starting pressure	initial pressure used to calculate the initial activities (initialpressure)
minimum pressure	minimum pressure within the uterus (minpressure)
maximum burst duration	maximum # time iterations for each burst (timeburst)
refractory duration	maximum # time iterations of the refractory period (timerefractory)
Action potential multiplier	factor that increases act(i,j) during an action potential (burstmultiplier)
refractory multiplier	multiplier used when tissue is in refractory period (refractorymultiplier)
weibullvar1	shape of the Weibull distribution for anatomy sensitivity (anatomysens)
weibullvar2	scope of the Weibull distribution for anatomy sensitivity
weibullvar3	start of the Weibull distribution for anatomy sensitivity
weibullvar4	shape of the Weibull distribution for action potential threshold (burstthreshold)
weibullvar5	scope of the Weibull distribution for action potential threshold
weibullvar6	start of the Weibull distribution for action potential threshold
**Calculation/output variables**	
pressure	intrauterine pressure at each time step
act(i,j)	contractile activity of the i,j region at a given time
activity(i,j,t)	the tensor of act(i,j), remembers all act(i,j) at all times
anatomysens(i,j)	sensitivity of the i,j region because of physical shape
anatomysenstensor(i,j,t)	the tensor that holds anatomysens(i,j)
burstthreshold(i,j)	action potential threshold for the i,j region
burst(i,j)	applies burstmultiplier if above threshold
refractoryfactor(i,j)	applies refractorymultiplier if in refractory period
refractory(i,j,t)	the tensor that holds refractoryfactor(i,j)
pressurevector(t)	the vector that remembers the pressure at all times

X.S1YYY: weibullvar1/weibullvar2/weibullvar3; S2ZZZ: weibullvar4/weibullvar5/weibullvar6.

Here X represents a specific set of input variables for row, column, timesteps, initial pressure, minimum pressure, maximum burst duration, refractory duration, action potential multiplier, and refractory multiplier (see Table 2). S1YYY refers to the anatomy sensitivity (anatomysens) seed value between 1000 and 1999. S2ZZZ refers to the action potential threshold (burstthreshold) distribution seed value 2000 through 2999. Weibull1 to 3 refer to the Weibull parameters for anatomy sensitivity and Weibull4 to 6 for action potential threshold parameters.

**Table 2:** Values for input settings.

Input set # (x)	Rows/columns/timesteps	Starting P/minimum P	Burst/refractory duration	AP/refractory multipliers
1	4/4/300	0.5/0.5	10/20	3/0.2
2	4/4/300	0.5/0.5	10/20	**2**/0.2
3	4/4/300	0.5/0.5	10/20	**1.5**/0.2
4	5/5/300	0.5/0.5	8/26	1.5/0.2
5	5/5/300	0.5/0.5	8/26	**2**/0.2
6	5/5/300	0.5/0.5	8/26	**4**/0.2
7	5/5/300	0.5/0.5	8/26	**8**/0.2
8	5/5/300	0.5/0.5	10/12	3/0.2
9	1/1/300	1/0.6	10/20	3/0.2
10	1/1/300	1/**0.55**	10/20	3/0.2
11	1/2/300	0.5/0.5	10/14	3/0.1
12	1/2/300	**1**/0.5	10/14	3/0.1
13	1/2/300	0.5/0.5	10/**10**	3/0.1
14	**i**/**j**/300	0.5/0.5	10/20	2/0.2

The format for reporting input values is:

x.S1yyy: weibullvar1/weibullvar2/weibullvar3; S2zzz: weibullvar4/weibullvar5/weibullvar6.

x is input set number; yyy and zzz refer to seed values between 000 and 999 (these are specific to Mathematica) and are used to generate the pseudorandom numbers. Pseudorandom numbers are used to select the specific values from the Weibull distributions that are assigned to each region.

Weibull distributions for anatomy sensitivity are generated from weibullvar1–3 and distributions for action potential threshold from weibullvar4–6.

Values in **bold** represent the changes made within the series.

## Results

**Figure 3:**
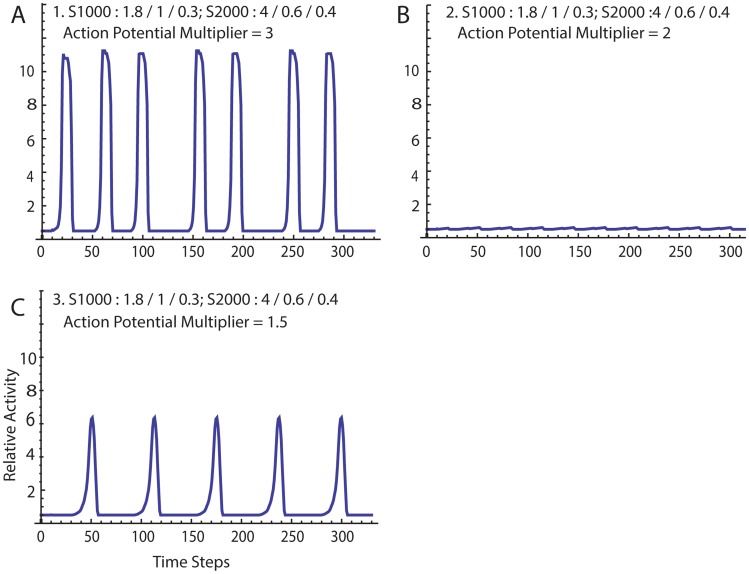
A. Simulation results using the default values. See Table 2 for complete set of input values. B. The action potential multiplier was changed to 2 from 3, simulating nifedipine exposure (all other input values remained the same as in A). C. The action potential multiplier was reduced further to 1.5, simulating increasing the nifedipine concentration.

Here we describe the behavior of the simulation for specific input values (Table 2). In figure 3A the default values, 1.S1000:1.8/1/0.3; S2000:4/0.6/0.4, reveal a pressure profile suggestive of patterns commonly seen using pressure catheters on women in labor. Contractions are regular, although show some variability of the period between contractions. Peak heights vary slightly. Each contraction arises spontaneously. Pressure rises slowly at first, then accelerates. There is a pseudo-plateau, then a pseudo-symmetrical falling phase. Of note, the refractory time is set at 20 iterations, yet the number of steps between some contractions is ∼40.

In figure 3B, the action potential multiplier is reduced to 2 from 3. Only small localized contractions are seen and coordinated organ-level contractions are lost. This behavior is what might be anticipated following exposure to nifedipine, which blocks L-type calcium channels and inhibits contractions in tissue strips in a dose-dependent manner [20]. However, reducing the action potential multiplier further to 1.5 (simulating increasing the concentration of nifedipine) results in a paradoxical reappearance of coordinated contractions (Fig. 3C).

**Figure 4:**
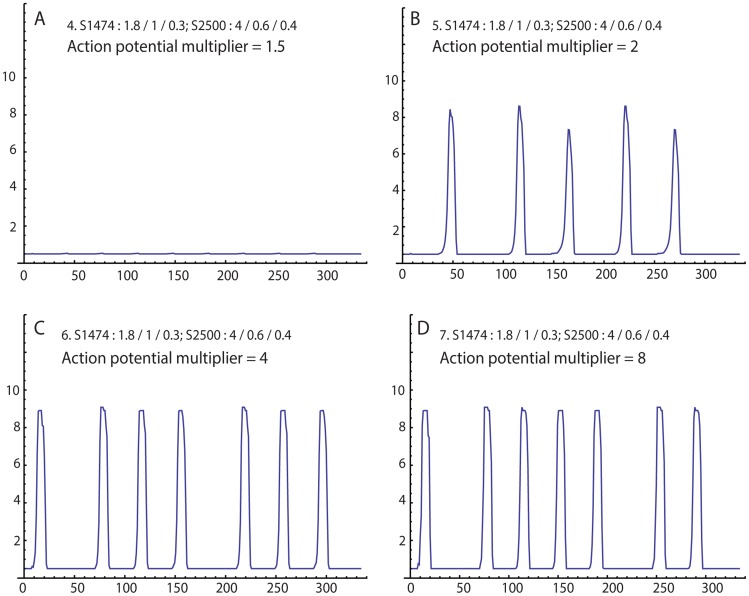
Simulation of a different “patient” using different seed values for anatomy sensitivity (1474) and action potential threshold (2500). Action potential multiplier is changed from 1.5 (A), to 2 (B), to 4 (C), to 8 (D), simulating increasing exposure to oxytocin.

In isometric contractility experiments, the primary effect of oxytocin exposure is an increase of force production. To simulate this effect, we step-wise increased the action potential multiplier from 1.5 to 2, then 4, then 8 (Fig. 4A, B, C, D, respectively). We also simulate a different patient by increasing the number of regions (using 5 rows and 5 columns) and changing the pseudorandom seeds, which assigns different anatomy sensitivity and action potential threshold matrices. When the action potential multiplier is 1.5, no coordinated contractions are seen. Increasing this value to 2 resulted in the appearance of irregular and infrequent contractions. At 4, the contractions became more regular, with increased force and frequency. At these settings, the time between contractions is near the limit of the refractory duration. Doubling the action potential multiplier to 8 resulted in only subtle changes of the contraction pattern.

**Figure 5:**
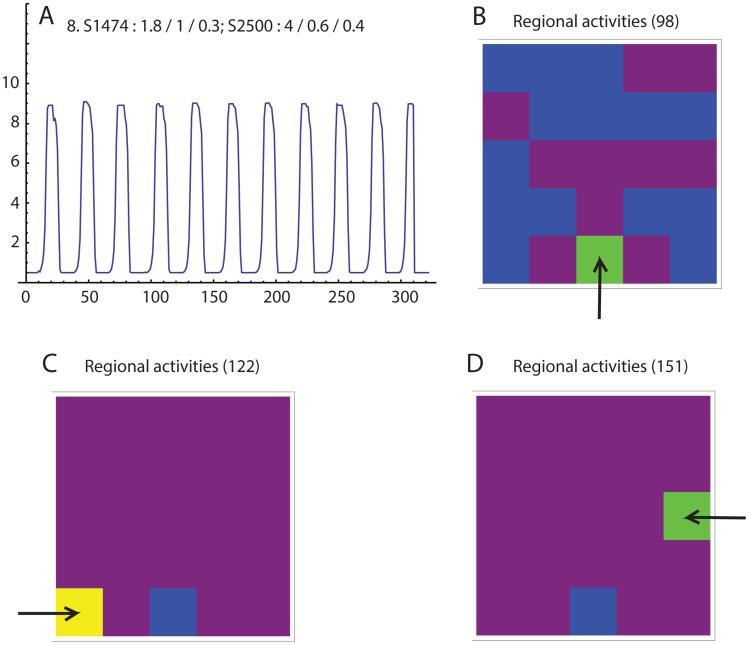
A. Simulation using the same seed values as in **figure 4**, and midrange parameter values. The periods between contractions varies only slightly. B, C, and D. Regional activities immediately prior to the 4^th^, 5^th^, and 6^th^ contractions, respectively. Each of the three contractions has a different apparent pacemaker (arrow).

Next we demonstrate that it is possible for the simulation to generate contractions that are initiated by multiple pacemakers. In this run, the rows and columns are set to reflect 25 regions (rows = columns = 5) and the random number seeds for anatomy sensitivity and action potential thresholds are set to 1474 and 2500, respectively. Regular contractions are seen with only slight variations of the interval between contractions (Fig. 5A. The color animation of the regional activities reveals that the first region to demonstrate significant activity that continues through a global contraction is not always the same (Fig. 5 B, C, D).

The likelihood that a region becomes active is increased with a high value of anatomic sensitivity, but a low value of action potential threshold. Therefore, to approximate the overall, or “total”, sensitivity of each region, we divided the anatomic sensitivity by the action potential threshold for each region. The most sensitive region is in row 5, column 1 (total sensitivity = 4.003). The second most sensitive region is in row 3, column 5 (total sensitivity = 2.024), and the third is in row 5 column 3 (total sensitivity = 1.975). The pacemaker for the 4^th^ contraction (Fig. 5B, Step 98) is the region in row 5, column 3, the pacemaker for the 5^th^ contraction (Fig. 5C, Step 122) is the region in row 5, column 1, and the pacemaker for the 6^th^ contraction (Fig. 5D, Step 151) is the region in row 3, column 5.

**Figure 6:**
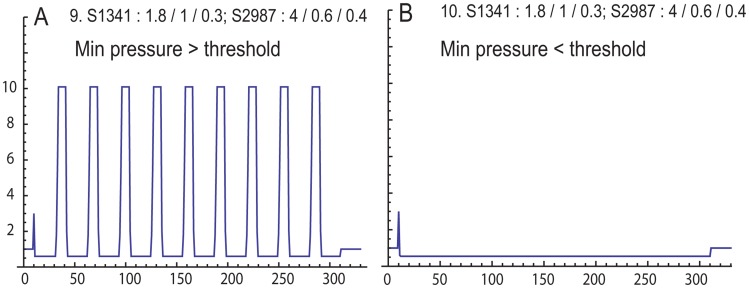
Simulation of single tissue, isometric contractility experiment. Seeds and Weibull parameters were specifically selected for the anatomy sensitivity to be near 1 (0.991), and the action potential threshold to be low (0.558). A. Minimum pressure set above action potential threshold (0.6) reveals repetitive contractions. B. Minimum pressure set below threshold (0.55) reveals no repetitive contractions, even though the initial pressure is above threshold.

An isolated tissue strip can be simulated with rows = columns = 1 (Fig. 6). Here we specifically set the anatomy sensitivity to a value near 1 (0.991, using anatomy seed 1341), since under isometric conditions the anatomy sensitivity equals 1 exactly. The value for threshold is 0.558 (threshold seed 2987). Repetitive trials reveal that the tissue will only contract if the minimum pressure is above 0.6, even if the initial pressure is above threshold (Fig. 6B). Therefore, minimum pressure is the key factor for determining if regional contractions will occur.

**Figure 7:**
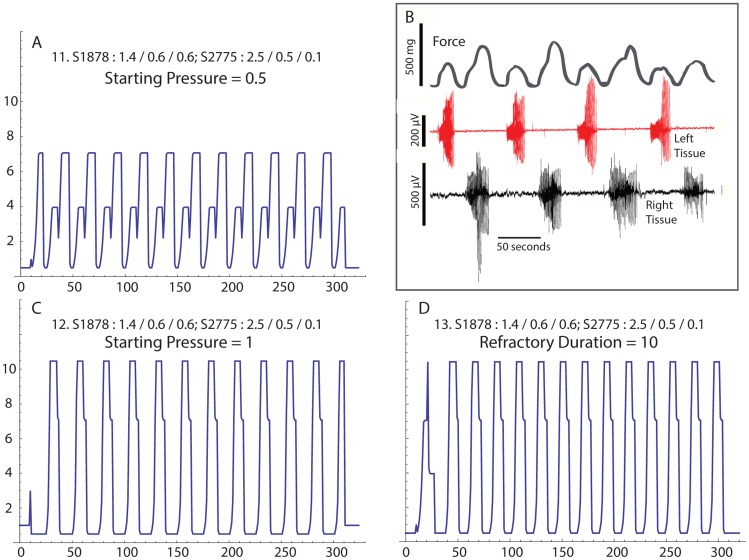
Two tissues linked end-to-end in isometric contractility experiment. A. Simulation where both tissues express repetitive contractions, but the tissues are contracting out-of-phase. B. Out-of-phase contractions experimentally recorded from two rat myometrial tissue strips demonstrating alternating contraction pattern corresponding to A (from ref. 16). “L” is the bioelectrical activity of the left tissue strip; “R” is the bioelectrical activity of the right tissue strip. C. Simulation after increasing the starting pressure, but keeping all other input values the same as in A, reveals in-phase contractions. D. Returning the starting pressure to the value in A, but decreasing the refractory duration also couples the tissue into an in-phase pattern.

When rows = 1, columns = 2 there are two regions, which simulates two mechanically linked tissue strips (Fig. 7 [16]. The anatomy seed and Weibull parameters are set to reflect both tissues with similar anatomy sensitivity values near 1 (0.986 and 0.989, respectively). The left and right tissues have action potential thresholds of 0.428 and 0.670, respectively, and the minimum pressure is set to fall between these values (0.5). In Fig. 7A the tissues appear to oscillate out of phase. The activity animation reveals that with these settings, the two regions are not ever highly active simultaneously (not shown) – first the “left” tissue becomes active, and then the “right”. This pattern correlates well with experimental observations (Fig. 7B) [16]. However, when the simulation is run with the starting pressure increased to 1 (Fig. 7C), the regions oscillate at the same time and the expressed pressure is large. It is also possible to coordinate the contractions by reducing the refractory duration to 10 (Fig. 7D, the starting pressure was returned to 0.5).

**Figure 8:**
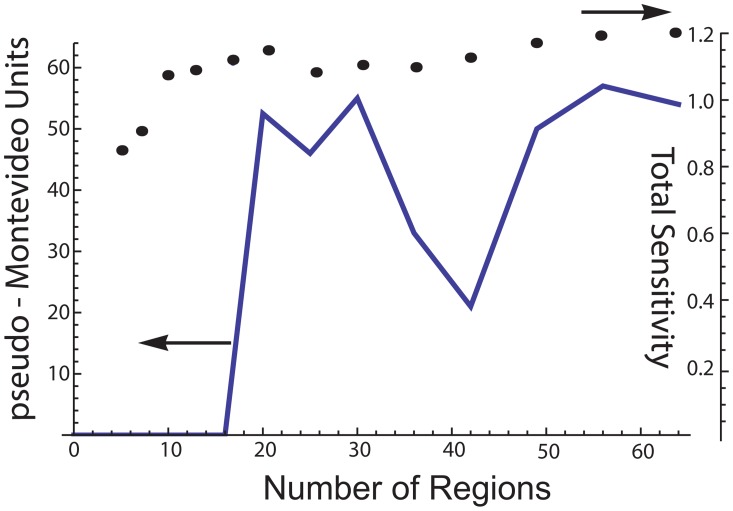
pseudo-Montevideo units are calculated from peak pressures and the total number of contractions expressed in 300 time steps. The number of rows (i) and columns (j) is varied, but otherwise the input values are the same as in Fig. 3B. As the total number of regions changes, the pseudo-Montevideo units (solid line) vary in a complex manner, expressing a peak between 18 and 30 regions. The mean of the total sensitivity (filled circles) is approximated from the anatomy sensitivity and the action potential threshold of all the regions. Because these values are pseudorandomly selected for each region, there is a slight variation of the mean total sensitivity, especially for 4 to 8 regions. But for 10 regions and above, the fall in pseudo-Montevideo units cannot be explained by changes of the total sensitivity.

In addition to the special cases where only one or two regions were simulated, changing the number of regions has important effects on the expression of contractions. To correlate the total force production as a function of the number of regions, we calculated pseudo- “Montevideo units” (pMV = peak force * # contractions in 300 time steps) and plotted this value as a function of the number of regions (Fig. 8). In figure 3B with 16 regions, reducing the value of the action potential multiplier to 2 from 3, the regional oscillations fail to create enough pressure to trigger a coordinated organ-level contraction. Continuing with these input values, global contractions are expressed when the number of regions is increased to 18 (6 rows, 3 columns), and the pMV units remain high through 30 regions. At 32 regions, the pMV units drop, and remain low through 42 regions. When the number of regions is 49 and above, the Montevideo units again increase. To ensure that the fall of force production between 32 and 42 regions was not the result of a large change of sensitivity as the number of regions varied, for each run we averaged the total sensitivity of all the regions to obtain the mean total sensitivity. While mean total sensitivity varies slightly, the onset of global contractions at 18 regions and the mid-range dip of force production at 32 to 42 regions does not appear to be attributable to changing sensitivities.

## Discussion

Our model is specifically limited to human labor because the human uterus is primarily a pressure-generating organ, and this may not be true for rodent or other lower animals. The two primary goals of this simulation are 1) to determine if our model of synchronization of regional contractions using pressure-tension mechanotransduction can reproduce the time-dependent profile of the uterine contractions of labor; and 2) to gain insight as to how cellular- and tissue-level physiology integrates into organ-level contractility. We demonstrate the general appearance of the pressure profile of the contracting uterus using a variety of input values, and therefore satisfied the first goal. The remainder of the discussion addresses the second goal.

The Law of Laplace strictly applies only to very thin-walled vessels, and numerical methods are available to accommodate thick walls and more closely quantify pressure-tension relationships. This is required for the heart, but the uterus does not express complex anatomy, an orderly mechanism for tissue recruitment, or large changes of volume or wall thickness with each contraction. Thus, while a more complex model would likely more closely describe the time course of the pressure rise as a function of each regional contraction, we found the Law of Laplace performs well and explains many key elements of human uterine function.

In our model we use the term “minimum pressure” to refer to the pressure between contractions, or clinically the baseline pressure from IUPC recordings. Between contractions, the resting passive activity (“resting tension”, or “tone”) of each region of the uterine wall is determined by multiplying by the anatomy sensitivity as dictated by the Law of Laplace. It is then the relationship between the resting tension and the threshold sensitivity that determines if any region will experience an action potential and contract (Fig. 6). Hence, the three dominant factors that determine if labor contractions will occur are the baseline pressure, the anatomy of the uterus, and the electrical sensitivity of each region. While myometrial electrical excitability properties have long been appreciated as key to the onset of labor, it is only recently that myometrial tone coupled with anatomy considerations [18] has been proposed to contribute to the onset of labor.

Under some conditions, regional oscillations spontaneously occurred, but failed to recruit enough regions to express what would reasonably be described as globally coordinated, or organ-level, contractions. Conditions could also be found where the opposite occurred, and organ-level contractions always arose from one or two spontaneous regional contractions. Under other conditions, both poorly coordinated and globally coordinated regional contractions were observed together.

There are two patterns of interactions that occur when regional activity is observed, but organ-level contractions are not expressed. First, a large proportion of the regions may have a low anatomic sensitivity or a high action potential threshold, and despite an underlying background of pressure rises, they are not recruited to participate in the contraction. This can be considered a “sensitivity failure”. Second, groups of the more sensitive regions can oscillate out-of-phase with other groups, which keeps pressures low. The blunted pressure development is responsible for the failure to recruit the intermediately sensitive regions. The second pattern can be considered a “synchronization failure”.

Highly interesting simulations are seen when poorly coordinated and organ-level contractions co-exist. In these cases, complex and emergent behavior result as input values are varied, and a great deal of insight is gained regarding the relationship between tissue-level and organ-level function. In figure 3A the gross appearance of the pressure profile suggests only coordinated contractions, but detailed inspection of the activity matrix show a few regions activate briefly between coordinated contractions. Nifedipine acts in a dose-dependent manner to reduce peak force in isometric myometrial strip experiments [20]. To approximate the L-type calcium channel blocking effects of nifedipine, we modestly reduce the action potential multiplier, and observe loss of the coordinated contractions (Fig. 3B). However, as we further reduce the action potential multiplier, coordinated contractions reappear (Fig. 3C), albeit with reduced frequency and strength when compared with the starting value. Thus, our simulation implies that greater concentrations of nifedipine, may under some conditions, actually be less effective at stopping organ-level contractions than lower doses. Clinically, nifedipine does exhibit variable responses as a tocolytic agent, and this simulation may assist with understanding this complex behavior.

In figure 4 we simulate the uterine response to oxytocin exposure by increasing the action potential multiplier. Beginning from conditions where no coordinated contractions are observed, simulating increasing oxytocin resulted in the appearance of contractions by overcoming sensitivity failure. This was followed by an increase of the peak force and frequency. By comparing the individual regional activities, the increases can be attributed to the larger action potential multiplier causing higher pressures from the first few regions that become activated. The higher pressures more rapidly recruit some regions that previously were out-of-phase. Hence, overcoming synchronization failure of the regional contractions increases contraction strength and frequency. Taken together, the simulation suggests that the organ-level effects of oxytocin result indirectly from cellular-level effects, but more directly from the complexity of the mechanotransduction component of our model.

At first impression, it is tempting to surmise that the most sensitive region will serve as a stable pacemaker, where the pacemaker is perhaps viewed as the first region to demonstrate activity and then participate in the contraction. However, this is not always the case. In figure 5, the most sensitive region (row 5, column 1) is nearly twice the sensitivity of the next most sensitive region. While this region directly initiates some contractions, other regions activate first for other contractions. Pace making responsibilities shift to these “alternate pacemakers” when the most sensitive region is in its refractory period and the majority of the other regions are not in theirs. This is an emergent property of a complex system, and in principle is not predictable. Therefore, a stable pacemaker is neither necessary nor sufficient for effective labor, and if our model withstands further investigation, the general concept of a pacemaker should be reexamined. (See “Rethinking the pacemaker concept”, below.)

Perhaps the most counterintuitive aspect of our model is that local variations of anatomy have significant bearing on the selection and distribution of pacemakers and the sequence of regional recruitment. While we used random sampling from a Weibull distribution to approximate these anatomic differences, it is possible for these values to be experimentally determined [2], and we eagerly await analysis of these data.

There are two, somewhat trivial, points regarding simulating a single region (Fig. 6; rows = columns = 1). First, in order to express oscillations, the minimum pressure must exceed the action potential threshold, the burst duration sets the width of the burst, and the refractory period duration sets the time between contractions. Thus the trivial second point: With only one region, and no possibilities for interactions with other regions, there is no possibility for appearance of emergent properties.

We have previously reported in two-tissue muscle bath studies that mechanotransduction alone can create an out-of-phase contraction pattern that we suggested was reminiscent of Braxton-Hicks contractions [16]. We simulate this experimental configuration using rows = 1 and columns = 2. We were successful in demonstrating that two interacting regions are capable of out-of-phase contractile patterns (Fig. 7A), although we are not able to identify initial conditions that simulated alternating contractions that were 180 degrees out-of-phase. The out-of-phase pattern we simulated was similar to the pattern we previously reported for two myometrial strips linked end-to-end (Fig. 7B) [16]. By doubling the starting pressure (0.5 to 1, Fig. 7C), the regions oscillated together, even though the input values that described the physiology of the regional interactions were identical. In this context the system appears to have a memory of the history of activity.

Returning the starting pressure to 0.5 and reducing the refractory duration to 10 also brings the tissue into a synchronized pattern (Fig. 7D). Thus, the relationship between the refractory period and the burst duration may cause one region to be in-phase or out-of-phase with the other. While this mechanism seem reasonable, it is likely that our model fails to include some important tissue-level mechanisms that may be required to fully explain *in vivo* behavior, such as out-of-phase synchronization. For example, under some conditions decreasing tissue tension may induce a contraction [21], or increasing tension may inhibit contractions [22].

Detailed investigation of the simulated contractile behavior as a function of the number of regions reveals that the expression and frequency of spontaneous organ-level contractions are dependent on the number of regions in a complex manner (Fig. 8). When there are a small number of regions (16 or fewer), it is unlikely that a spontaneous regional contraction occurs in an anatomical location that raises intrauterine pressure enough to trigger other regional contractions – a process we previously termed sensitivity failure. As the number of regions increases, coordinated contractions are expressed. However, further increasing the number of regions above 30 results in a decrease of the frequency of contractions, and pMV falls. This is because contraction of a small percentage of the available regions increases pressure only slightly, and groups of oscillating regions fail to coordinate – a process we previously termed synchronization failure. As the number of regions is increased past 42, contractions again become more frequent, and pMV returns to prior levels. This effect occurs because the additional regions include many that are critically sensitive to smaller pressure changes, which increases recruitment enough to overcome synchronization failure. However, more regions means smaller regions, which indicates poor electrical connectivity. This suggests that the contraction-associated proteins are not up-regulated, and tissue excitability and contractility is also poor. Taking this into consideration, Figure 8 suggests that 18 to 30 is the optimal number of regions for expression of coordinated contractions, which agrees well with our estimation from SQUID array data (see “additional details of the calculation”, point #1). Hence, in the context of our model, gap junctions are necessary for expression of coordinated uterine contractions because they establish the number of regions within a critical range. Too few, and the uterus cannot express emergent properties; too many, and contractions of a single region are unable to raise intrauterine pressure sufficiently to recruit other regions.

There are several limitations of our simulation that are more reflective of the assumptions we make regarding tissue-level physiology rather than specific limitations inherent to the model. For example, we assume that action potential bursts enhanced contractile activity via a multiplication factor (the action potential multiplier). It is likely, however, that electrical activity causes tissue contractions through a much more complex mechanism [2]. A similar criticism can be applied to our simulation of the refractory period, which could also be simulated by increasing the action potential threshold rather than stopping the burst and reducing the passive activity. Most importantly, we do not have detailed knowledge of how acutely increasing tissue tension induces a contraction – is it really depolarization above threshold? It should be emphasized that the tissue-level physiology used in the model is testable and we encourage refinements to the model based on new knowledge.

### Explaining large uterine action potential speeds

It is possible to measure the apparent speed that an action potential propagates through the human uterus using surface EMG. Two or more pairs of EMG electrodes are placed on the abdominal surface, then the distance between the electrode pairs is divided by the time between the onset of electrical activity. Speeds have been reported to average in excess of 50 cm/sec [4], which is nearly more than an order of magnitude greater than the speeds measured in rodent *in vitro* [11]. This discrepancy has been explained [5] by assuming the tissue-level action potential travels long distances at much slower speeds, but they are measured as faster because the initiation point is presumably in between or lateral to the electrodes. In our model, a single action potential does not travel great distances. We propose that high speeds are artifacts of measurement that arise when electrodes record from two regions that are separated by a relatively long distance, and the regions are independently recruited by mechanotransduction at nearly the same time because the regions sense the same intrauterine pressure.

Recently, however, action potential propagation velocities (speed and direction) were measured in term pregnant women in labor [23]. Using a 2-dimensional array over a 14 cm×14 cm grid of electrode pads, the average speed over 35 contractions was 2.18±0.68 cm/sec. The maximum measuring distance was 17.5 cm on the diagonal, but only 3 velocities were measured near the diagonals. Most measurements were over distances between 3.5 and 7 cm. From our model this most likely represents measuring the action potential propagation velocity within one region.

### The myometrial myogenic response

In small arteries, pressure-dependent contractions regulate local blood flow by what is referred to as the myogenic response [17]. In brief, acutely increasing intravascular pressure results in acute contraction of the artery wall, which narrows the lumen and helps maintain the flow of blood through the artery at a constant rate. In our model of the laboring uterus, acutely raising intrauterine pressure results in an acute contraction of the uterine wall, which further increases pressure and recruits more regions to participate in the contraction. In this sense, the physiological process we propose for the uterus closely parallels the myogenic response of the artery, with the exception that the artery contraction is tonic and the myometrial contraction is phasic.

Despite this difference, we propose that the term “myometrial myogenic response” should be used to refer to the process of generating large intrauterine pressures by synchronizing uterine wall contractions via mechanotransduction. In a more limited sense, it can also be used to describe the mechanism of stretch-initiated contractions. The key purpose of introducing this terminology is to help differentiate the mechanisms used to rapidly coordinate uterine contractions from the mechanotransduction mechanisms used to regulate gene expression over longer time frames.

### Applying cellular automata simulations to complex biological systems

Elementary CAs are well-defined mathematical constructs previously used to investigate the emergent properties of complex systems [24]. There are only 256 possible rules and Wolfram places each into one of four classes based on the expression of complex behavior. Starting values are not considered in the classification of the rule. However, biological CAs are quite different from elementary CAs. Rules are layered to integrate lower level processes into higher level function, and they are highly constrained (or perhaps enriched) by the physiology. After establishing the rules, the main purpose of the biological CA is to examine effect of the input values on the CA behavior, and determine how closely the higher level function is simulated.

Hence, a great deal of insight can be gained on the relationship of less complex physiology to more complex biological behavior, and it is worthwhile to classify biological CAs. Therefore, we propose that the input values form the basis for classification of biological CAs. The “input-based classification” system should parallel the rule-based classification established by Wolfram. A set of input values that converges to a uniform state (no change of activity over time) is class 1. An input value set that converges to a repetitive, or stable state is class 2. A class 3 set yields a “chaotic” state, without repeating patterns or large structures. A class 4 set generates complex behavior where emergent global patterns occur.

In addition, there must be two constraints on biological CAs if they are to be classifiable and yield input-based classes. First, the rules must be consistent with known relevant physiological processes, although the rules do not need to describe all known processes. For example, a CA of the pregnant uterus should not contain a rule describing long range efferent neural connections (which are known to not exist). Yet it is not necessary that it contain a rule describing prostaglandin paracrine signaling, even though the physiology is well-known. Furthermore, using a rule that describes a process that is not clearly a “known relevant physiological process”, must be described as such, and input values should allow testing of the rule. It could be argued that our assumption that there are limits to the distances action potentials can propagate in the human uterus is a speculation. But since we test this by allowing the numbers of regions to vary, the CA remains classifiable.

Second, the input values must be physiologically reasonable and potentially measureable, or calculable, from experiments. In formulating the rules, each input variable should be aligned as closely as possible with a measurable physiological effect. This will allow the CA to test how modifying the physiology changes function. Lastly, in order to be assigned to a class, the input values must be physiologically reasonable. As an example for this CA, any set of input values that contains 0 for the minimum pressure cannot be assigned to a class, since the uterus always maintains a non-zero baseline pressure.

Using these definitions, an example of class 1 behavior is figure 6B. An example of class 2 behavior is figure 6A. Figure 3A (the default input set) at first glance seems to be class 2, but because of the variation of the interval between contractions, is best placed in class 4. Although not shown, a more obvious example of class 4 behavior occurs with input set 4/4/300/0.5/0.7/8/24/3/0.2.S1246:1.8/0.6/0.55;S2648:4/0.8/0.1. We were unable to find a set of input values that displayed class 3 behavior, which may be a reflection of the stability of the underlying physiological system.

In addition to classes, there should be some notation regarding the relationship between the input values and the physiological relevance of the outcome. This will vary based on the biological system. For example, a uniform state without global activity (e.g. class 1) aptly describes the uterus for much of the pregnancy, and examining the range of input values where this occurs is important. In other biological systems this uniform state may be physiologically less relevant (perhaps a CA describing brain activity). Therefore we propose that for biological CAs an additional modifier may be added based on the relevance to function. First we propose the “N” modifier, which refers to behavior observed within the range of normal functioning. The “X” modifier, refers to behavior never observed under any conditions, and the “P” modifier for behavior with less than normal function (i.e. pathological). For behavior at the transition between states, for example between normal and pathological states, the N-P descriptor could be used. Ultimately we seek to classify and study patterns of input sets that will help determine the relationship between the physiology of the components and the complex and emergent behavior of the organ. Using this terminology, the default input value set (Fig. 3 A) is class 4-N. Previous mathematical simulations of uterine function have seemed focused on identifying conditions that yield repetitive phasic contractions, or class 2-N. Our work suggests that forcing class 2 behavior of closed-form models may not be necessary, and may artificially narrow the boundary conditions.

### Rethinking the pacemaker concept

As discussed above, this CA offers an explanation for why a stable pacemaker has not been found in the human uterus, and we suggest that the concept of a pacemaker of the uterus should be reexamined. If the framework of our model is correct, the mode of operation of normal labor exploits the emergent properties of a complex system (class 4). Yet at any point in time there will be one region with the largest total sensitivity. That region can be reasonably called *the* pacemaker because the cascading events that create each contraction are largely driven by the activity of that region. By this definition, the pacemaker only needs to initiate the events that eventually progress to generate repetitive coordinated contractions. It does not need to be the trigger at the leading edge of every contraction (Fig. 5), or even participate in every contraction. Previously, attempting to identify the uterine pacemaker involved looking for the first activity at the beginning of every contraction, but we propose that looking for the region that expresses the highest frequency of activity will identify the pacemaker. This is a particularly difficult task in practice, since current EMG techniques, and even the SQUID array, are able to examine only the front wall of the human uterus.

As long as there are no changes that effect the total sensitivity of the regions, it is likely that the pacemaker location will be relatively stable, perhaps over many contractions. However, the pacemaker location will change as the anatomy or excitability properties of the uterus as a whole change. Specifically, the pacemaker site will be highly dependent on tissue-level electrical interconnectivity. This is because changing the size of the regions changes two factors: the local anatomy, and (as we demonstrate in figure 8) the interactions of the regions in a class 4 system.

It may be possible to observe repetitive regional activity that fails to recruit other regions and is not associated with coordinated contractions. That behavior would likely be class 2. As we proposed above, true labor arises out of class 4 behavior, so repetitive activity associated with class 2 behavior that fails to yield coordinated contractions is merely repetitive activity and not pacemaker activity.

### Clinical applications

The simulation at this stage of development is not adequate to observe clinical tracings and then suggest specific treatments designed to normalize abnormal patterns. However, this is a long-range goal, and we encourage continued investigation and further refinement of the model by others. To this end, we provide the code and documentation for this simulation in supplementary information S1 and S2. The program is free to download, modify, and investigate.

In conclusion, simulation of our model successfully links cellular- and tissue-level physiology to observable organ-level functioning. This success supports our model of electrical activity and mechanotransduction synergistically combining into a dual mechanism of global uterine function. If this model withstands further investigation, we propose that the concept of a stable uterine pacemaker triggering and participating each contraction be abandoned for human labor. In our model, the distances action potentials can propagate *in vivo* define the size of the functional regions, and the number of functional regions is important in the expression of coordinated contractions. We propose that further work is needed to determine how these distances vary with different clinical conditions, including multiple gestation, uterine anomalies, mass lesions of the uterus, and gestational age.

## Supporting Information

Supporting Information S1 The simulation written in Mathematica, Version 9 (requires program to open). Parameter names are detailed in the text.
(NB) Click here for additional data file.

Supporting Information S2 The simulation in PDF format (does not require Mathematica to open).
(PDF)
