# Supplementary material for: Linking Myometrial Physiology to Intrauterine Pressure; How Tissue-Level Contractions Create Uterine Contractions of Labor
Source: PLoS Comput Biol. 2014 Oct 16;10(10):e1003850. doi: 10.1371/journal.pcbi.1003850 (PMC4199482; doi:10.1371/journal.pcbi.1003850)
Supplement: Supporting Information S2 — The simulation in PDF format (does not require Mathematica to open). (PDF) [file pcbi.1003850.s002.pdf]

```
ClearAll[anatomysens, anatomysenstensor, act, activity, burst,
  burstthreshold, burstthresholdtensor, refractory, pressurevector];

SetAttributes[{anatomysens, anatomysenstensor, act, activity, burst ,
   burstthreshold, burstthresholdtensor, refractory, pressurevector}, Listable];
Print["Use default values or customize your own?"];
(*Begin input area*)
Default[f] = default; (*The beginning calls the default values for parameters*)
f[d_.] = d;
d = f[];
Control[{d, {default, customize}}]
(*Default input parameter area*)
If[d == default,
  rows = 4; columns = 4; (*rows and columns define the number
   of regions and is the 'size' of the uterus, range 3 to 6*)
  timesteps = 300;      (*# iterations of the simulation,
  50 to 300.  Each step is approximately 5 seconds*)
  initialpressure = .5;   (*Used to initialize arrays. Choose
    any value between 0 and 10. >5 to start with a contraction*)
  minpressure = .5;   (*This is the tone of the relaxed uterus;
  value 0.2 to 0.7;  Maxpressure is ~10; Maxactivity of any region is 10*)
  timeburst = 10;          (*this is the maximum time
   span for an Action Potential burst,range 5 to 15*)
  timerefractory = 20;    (*the time a region is refractory
   following a burst lasting the full timeburst, =5 to 30*)
  burstmultiplier = 3;    (* The multiplier on contractile
   activity used when an action potential is expressed*)
  refractorymultiplier = .2; (*the multiplier that reduces
   activity when a region is refractory, range 0 to .5*)
  (*Pseudo-Random number generation parameters. Weibull
    distributions are used to calculate the anatomy and
    electric sensitivities.  var1 and 4 are shape parameters;
  var2 and 5 are scale parameters, lower value yields sharper peak;
  var3 and 6 set lowest values of distributions*)
  random1 = 1000; random2 = 2000;
  (*Default seed values for anatomy and threshold distributions*)
  weibullvar1 = 1.8; weibullvar2 = 1; weibullvar3 = .3; (*Anatomy;
  var1: 1.2 to 3;  var2: 0.4  to 1.2; var3: 0.1 to .6 *)
  weibullvar4 = 4; weibullvar5 = .6; weibullvar6 = .4]; (*Threshold;
var4: 2 to 8;  var5: 0.1 to 1;    var6: 0.1 to 1 *)

(*custom input parameter area*)
{Slider[Dynamic[rows], {1, 8, 1}], Dynamic[rows], "Rows"}
{Slider[Dynamic[columns], {1, 8, 1}], Dynamic[columns], "Columns"}
{Slider[Dynamic[timesteps], {50, 300, 1}],
 Dynamic[timesteps], "Time steps, each step is ~5 seconds"}
{Slider[Dynamic[initialpressure], {.5, 5, .5}],
 Dynamic[initialpressure], "starting pressure"}
{Slider[Dynamic[minpressure], {.2, .7, .05}],
```

```
 Dynamic[minpressure], "minimum pressure"}
{Slider[Dynamic[timeburst], {5, 15, 1}],
 Dynamic[timeburst], "Maximum burst duration"}
{Slider[Dynamic[timerefractory], {5, 30, 1}],
 Dynamic[timerefractory], "refractory duration"}
{Slider[Dynamic[burstmultiplier], {1, 10, .5}],
 Dynamic[burstmultiplier], "Action Potential Multiplier"}
{Slider[Dynamic[refractorymultiplier], {0, .5, .05}],
 Dynamic[refractorymultiplier], "refractory multiplier"}

ttimesteps = timesteps + timeburst + timerefractory;
(*used to size tensor calculations correctly*)
counter = 0;     (*a dummy counter used in the burst calculation*)
(*establish sizes and initial values of matrices and tensors*)
act = Table[0, {i, rows}, {j, columns}];
activity = Table[1, {i, rows}, {j, columns}, {k, ttimesteps}];
anatomysens = Table[1, {i, rows}, {j, columns}];
anatomysenstensor = Table[1, {i, rows}, {j, columns}, {k, ttimesteps}];
burst = Table[1, {i, rows}, {j, columns}, {k, ttimesteps}];
burstthreshold = Table[.5, {i, rows}, {j, columns}];
burstthresholdtensor = Table[1, {i, rows}, {j, columns}, {k, ttimesteps}];
pressurevector = Table[initialpressure, {k, ttimesteps}];
refractory = Table[1, {i, rows}, {j, columns}, {k, ttimesteps}];

(*Establish the anatomy and electric sensitivity values from pseudo-
 random Weibull distributions*)
Print["↓↓↓ Anatomic sensitivity distribution ↓↓↓"];
{Slider[Dynamic[weibullvar1], {1.2, 3, .1}],
 Dynamic[weibullvar1], "anatomy distribution curve shape"}
{Slider[Dynamic[weibullvar2], {.4, 1.2, .1}],
 Dynamic[weibullvar2], "anatomy scope"}
{Slider[Dynamic[weibullvar3], {.1, .6, .05}], Dynamic[weibullvar3], "anatomy low"}
{Slider[Dynamic[random1], {1000, 2000, 1}],
 Dynamic[random1], "Anatomy pseudorandom number generator seed"}
SeedRandom[random1];
anatomysens = RandomVariate[
   WeibullDistribution[weibullvar1, weibullvar2, weibullvar3], {rows, columns}];
Plot[PDF[WeibullDistribution[weibullvar1, weibullvar2, weibullvar3], x],
 {x, 0, 4}, ImageSize → Small, Filling → Axis, PlotRange → All]
Print[anatomysens // MatrixForm];
Print["↑↑↑ Anatomic sensitivity distribution ↑↑↑"];
Print["↓↓↓ Action Potential threshold distribution ↓↓↓"];
{Slider[Dynamic[weibullvar4], {2, 8, .5}],
 Dynamic[weibullvar4], "threshold distribution curve shape"}
{Slider[Dynamic[weibullvar5], {.1, 1, .1}],
 Dynamic[weibullvar5], "threshold scope"}
{Slider[Dynamic[weibullvar6], {.1, 1, .1}],
 Dynamic[weibullvar6], "threshold low value"}
{Slider[Dynamic[random2], {2000, 3000, 1}], Dynamic[random2],
```

```
 "Action potential threshold random number generator seed"}
SeedRandom[random2];
Plot[PDF[WeibullDistribution[weibullvar4, weibullvar5, weibullvar6], x],
 {x, 0, 2}, ImageSize → Small, Filling → Axis, PlotRange → All]
burstthreshold = RandomVariate[
   WeibullDistribution[weibullvar4, weibullvar5, weibullvar6], {rows, columns}];
Print[burstthreshold // MatrixForm];
Print["↑↑↑ Action Potential threshold distribution ↑↑↑"];

(*make arrays into tensors for use in calculations
 below.  Arrays are put into tensor to be identical at each time*)
Do[anatomysenstensor[[i, j, k]] = anatomysens[[i, j]],
  {i, rows}, {j, columns}, {k, ttimesteps}];
Do[burstthresholdtensor[[i, j, k]] = burstthreshold[[i, j]],
  {i, rows}, {j, columns}, {k, ttimesteps}];

(*calculate the initial activity tensor based on initial conditions*)
activity = anatomysenstensor * initialpressure;
Do[
  (*check that the activity of each region is below 10*)
  Do[activity[[i, j, t]] = If[activity[[i, j, t]] > 10, 10, activity[[i, j, t]]],
   {i, rows}, {j, columns}];
  (*calculate the pressure vector based on initial conditions*)
  act = activity[[All, All, t]] / anatomysenstensor[[All, All, t]];
  pressure = Total[act, 2] / (rows * columns);
  pressurevector[[t]] = pressure
  , {t, ttimesteps}];
(*Begin the mechanotransduction calculation. Create the burst
 tensor. Stepwise calculate the activity tensor for all regions,
then calculate the pressure vector.  Note calculation starts
 at trefractory to accommodate going backwards in time*)
Do[
 Do[
  (*This section determines if each region is
   experiencing a burst and creates refractory periods if timeburst
   is reached.  This calculation is executed only if the tissue
   is not in the refractory period, i.e.refractory(i,j,t) = 1*)
  If[refractory[[i, j, t]] > .98,
   (*Successively calculate the burst tensor.  Initially it was set to unity,
   Check if activity>threshold*)
   burst[[i, j, t]] = If[activity[[i, j, t]] > burstthresholdtensor[[i, j, t]],
     burstmultiplier, burst[[i, j, t]]];
   (*Stop the burst if >burstthreshold activity existed
     over the prior timeburst steps*)
   Do[counter = If[activity[[i, j, t - n + 1]] > burstthresholdtensor[[i, j, t]],
      counter + 1, 0], {n, timeburst}];
   (*If suprathreshold activity for the prior timeburst steps,
   then counter = timeburst.  If true, dummy variable "test" goes to 1*)
   test = If[counter > (timeburst - .1), 1, 0];
```

```
    counter = 0;
    (*When test=1, make burst =
     1 and refractory = refractoryvalue for the next trefractory steps*)
    Do[burst[[i, j, t + n]] = If[test > .5, 1, burst[[i, j, t + n]]],
     {n, timerefractory}];
    Do[refractory[[i, j, t + n]] = If[test > .5, refractorymultiplier, 1],
     {n, timerefractory}]]
   , {i, rows}, {j, columns}];

 Do[
  (*This is the main calculation area for
   regional contractile activities and intrauterine pressure.
    Begin pressure calculation by extracting activity matrix for
   each region and correcting for its anatomical location*)
  Do[act[[i, j]] = activity[[i, j, t]] / anatomysenstensor[[i, j, t]],
   {i, rows}, {j, columns}];
  (*pressure is now the average activity(Laplace corrected) of each region
    at that time.  Note that restriction act(i,j)<10 does not apply,
  because this is the amount of contractile force that goes
   toward creating intrauterine pressure*)
  pressure = Total[act, 2] / (rows * columns);
  (*Check that the calculated pressure is greater than the minimum pressure*)
  pressure = If[minpressure < pressure, pressure, minpressure];
  (*the pressure vector remembers the pressure at each time*)
  pressurevector[[t]] = pressure;
  (*Calculate regional activities at the next time from the current pressure,
  burst, refractory and anatomy multipliers*)
  activity[[i, j, (t + 1)]] = pressure * anatomysenstensor[[i, j, t]] *
    burst[[i, j, t]] * refractory[[i, j, t]];
  (*check again that each activity is below 10 because this is
   the actual contractile activity the tissue generates*)
  activity[[i, j, (t + 1)]] = If[activity[[i, j, (t + 1)]] > 10,
    10, activity[[i, j, (t + 1)]]]
  , {i, rows}, {j, columns}]
 (*redo all calculations for the next time step*)
 , {t, timeburst, ttimesteps - timerefractory}]
(*End of mechanotransduction calculations*)

(*Output section*)
(*bypass 3-D plot for rows=columns=1*)
If[rows + columns > 2.2,
 ListPlot3D[activity, PlotRange → {0, 14}, ViewPoint → {0, -2, 1}]]
ListLinePlot[pressurevector, PlotRange → {0, 14},
 DataRange → {0, ttimesteps}, ImageSize → Large]
ListAnimate[Table[ArrayPlot[Round[activity[[All, All, t]]],
    ImageSize → Small, PlotLabel → "Regional activities "[t],
    ColorRules -> {10 → Red, 9 → Red, 8 → Magenta, 7 → Orange, 6 → Orange,
      5 → Yellow, 4 → Yellow, 3 → Green, 2 → Cyan, 1 → Blue, 0 → Purple}]
  , {t, 1, ttimesteps, 1}], AnimationRate → .75, AnimationRunning → False,
```

```
 DisplayAllSteps → True]
Graphics[{Purple, Rectangle[{0, 0}, {1, 2}], Blue,
  Rectangle[{1, 0}, {2, 2}], Cyan, Rectangle[{2, 0}, {3, 2}] , Green,
  Rectangle[{3, 0}, {4, 2}], Yellow, Rectangle[{4, 0}, {5, 2}], Yellow,
  Rectangle[{5, 0}, {6, 2}], Orange, Rectangle[{6, 0}, {7, 2}], Orange,
  Rectangle[{7, 0}, {8, 2}], Magenta, Rectangle[{8, 0}, {9, 2}], Red,
  Rectangle[{9, 0}, {10, 2}], Red, Rectangle[{10, 0}, {11, 2}]}]
Print["0 1 2 3 4 5 6 7 8 9 10"]
(*Print["activity tensor i,j,t  ",activity//MatrixForm];*)
(*Print["burst ",burstmultiplier//MatrixForm];*)
(*Print["refractory ",refractory//MatrixForm];*)
(*Print[pressurevector//MatrixForm]*)
```
